# Supplementary material for: Prostate-Specific Membrane Antigen (PSMA) Expression Predicts Need for Early Treatment in Prostate Cancer Patients Managed with Active Surveillance
Source: Int J Mol Sci. 2023 Nov 7;24(22):16022. doi: 10.3390/ijms242216022 (PMC10671119; doi:10.3390/ijms242216022)
Supplement: Supplementary file 1 [file ijms-24-16022-s001.zip › ijms-2655686-supplementary.pdf]

# Prostate Specific Membrane Antigen (PSMA) expression predicts need for early treatment in prostate cancer patients managed with active surveillance.

**Elham Ahmadi, et al.**

# Supplementary Figures and Table

**Figure s1: Markers expression in epithelial and stromal elements. A)** Representative images revealing distinct PSMA expression in prostatic epithelial cells, while no case of stromal PSMA expression was identified in this study. Note GLUT1 and ACLY expression in both epithelial and stromal elements (Dominant in epithelial cells and mild/scattered in stromal fibromuscular tissue). **B)** Nerve bundle in a prostate tissue biopsy (40X magnification), revealing strong GLUT1 (especially in perineurium) and ACLY expression, while they didn't show any degree of PSMA expression (Red arrows: Malignant glands, green arrows: Benign glands, yellow arrows: fibromuscular stromal cells, black arrows: nerve bundle, blue arrow: perineurium).

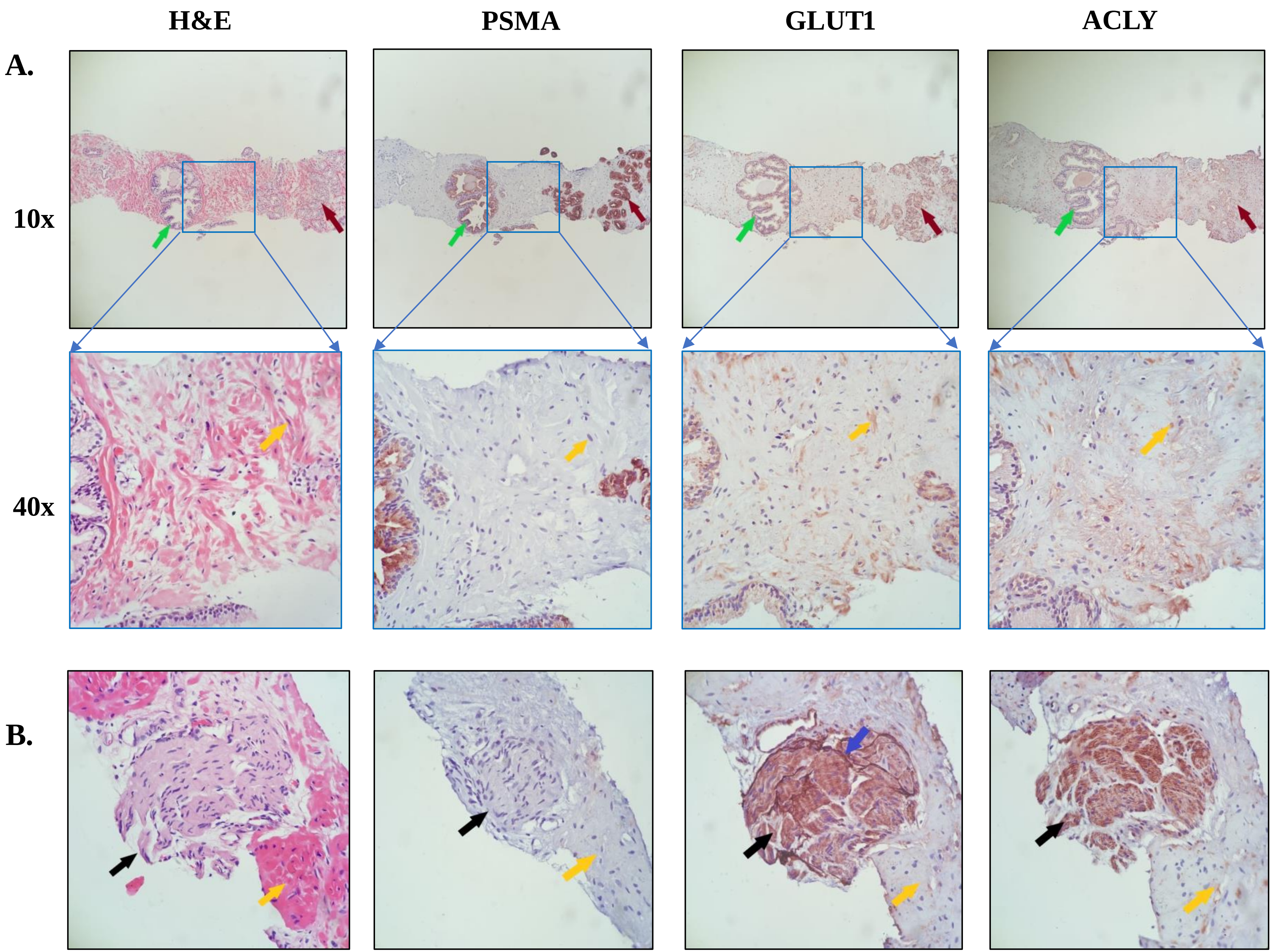

**Figure s2**

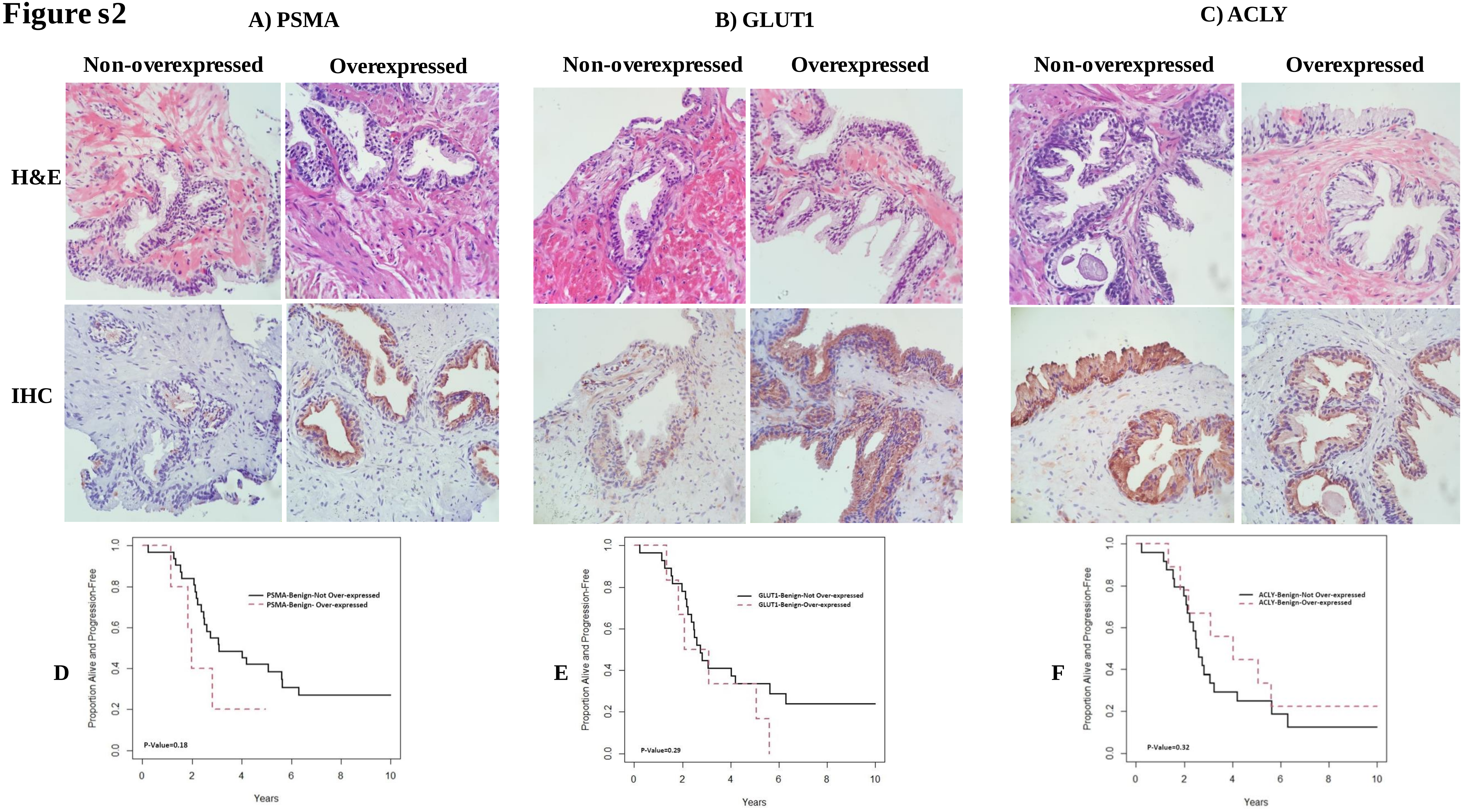
A) PSMA
B) GLUT1
C) ACLY
Non-overexpressed
Overexpressed
Non-overexpressed
Overexpressed
Non-overexpressed
Overexpressed
H&E
IHC
D
E
F
Proportion Alive and Progression-Free
Years
PSMA-Benign-Not Over-expressed
PSMA-Benign- Over-expressed
P-Value=0.18
GLUT1-Benign-Not Over-expressed
GLUT1-Benign-Over-expressed
P-Value=0.29
ACLY-Benign-Not Over-expressed
ACLY-Benign-Over-expressed
P-Value=0.32

**Figure s2. Correlation of markers expression in benign glands with time to treatment (TtT) in PC patients managed with AS program.** Images were taken at 40x magnification. **A)** Left images : 64 Y/O patient, GS=3+3=6 (ISUP grade group 1), revealing low PSMA expression in benign glands with a long time to progression (67.1 months). Right images: 66 Y/O patient, GS=3+3=6 (ISUP grade group 1), revealing PSMA overexpression in benign glands, who had a relatively short time to progression (14 months). **B)** Left images: 83 Y/O patient, GS=3+3=6 (ISUP grade group 1), revealing low GLUT1 expression in benign glands with a long time to progression (172.6 months). Right images: 83 Y/O patient, GS=3+3=7 (ISUP grade group 1), revealing GLUT1 overexpression in benign glands, who had a relatively short time to progression (37 months). **C)** Left images: 84 Y/O patient, GS=3+3=6 (ISUP grade group 1), revealing low ACLY expression in benign glands with 75.6-month time to progression. Right images: 64 Y/O patient, GS=3+3=7 (ISUP grade group 1), revealing ACLY overexpression in benign glands, with 67.4-month time to progression. **D, E and F)** Kaplan-Meier survival curves for PSMA, GLUT1 and ACLY expression in the benign component in PC patients managed with AS program. Patients with PSMA and GLUT1 over-expression in the benign component had an insignificantly increased rate of disease progression (P-Value= 0.18 and 0.29, respectively) compared to patients with PSMA and GLUT1 non-overexpression in benign components.

**Figure s3. Examples of different cytoplasmic intensities applied in the H-Score system.** Prostate biopsy tissue involved by prostate adenocarcinoma, IHC stained for PSMA marker (40X magnification), showing no expression (score 0), weak expression (score +1), moderate expression (score +2) and strong expression (score +3). Note apical accentuation in strong PSMA expression.

**Negative (0)** **Mild (+1)** **Moderate (+2)** **Severe (+3)**

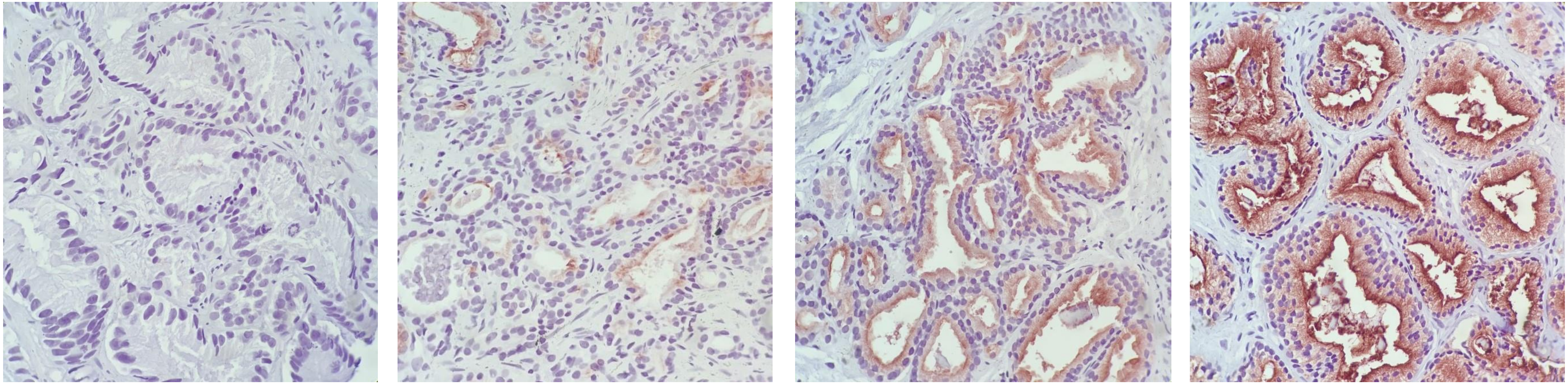

**Figure s4. Examples of AMACR IHC staining for confirmation of the malignant nature of glands, morphologically consistent with prostate adenocarcinoma** (Upper images: H&E staining, Lower images: AMACR IHC staining). **A)** A focus of prostate biopsy tissue (40x magnification) involved by a few infiltrative small acini surrounded by benign glands and epithelium, which malignant glands are positive for AMACR IHC staining (Red arrows: Malignant glands, blue arrows: Benign glands). **B)** A focus of prostate biopsy tissue (10x magnification) mostly involved by infiltrative small acini surrounded by scattered benign glands and epithelium, which malignant glands are positive for AMACR IHC staining (Blue arrows: Benign glands which are negative for AMACR IHC staining).

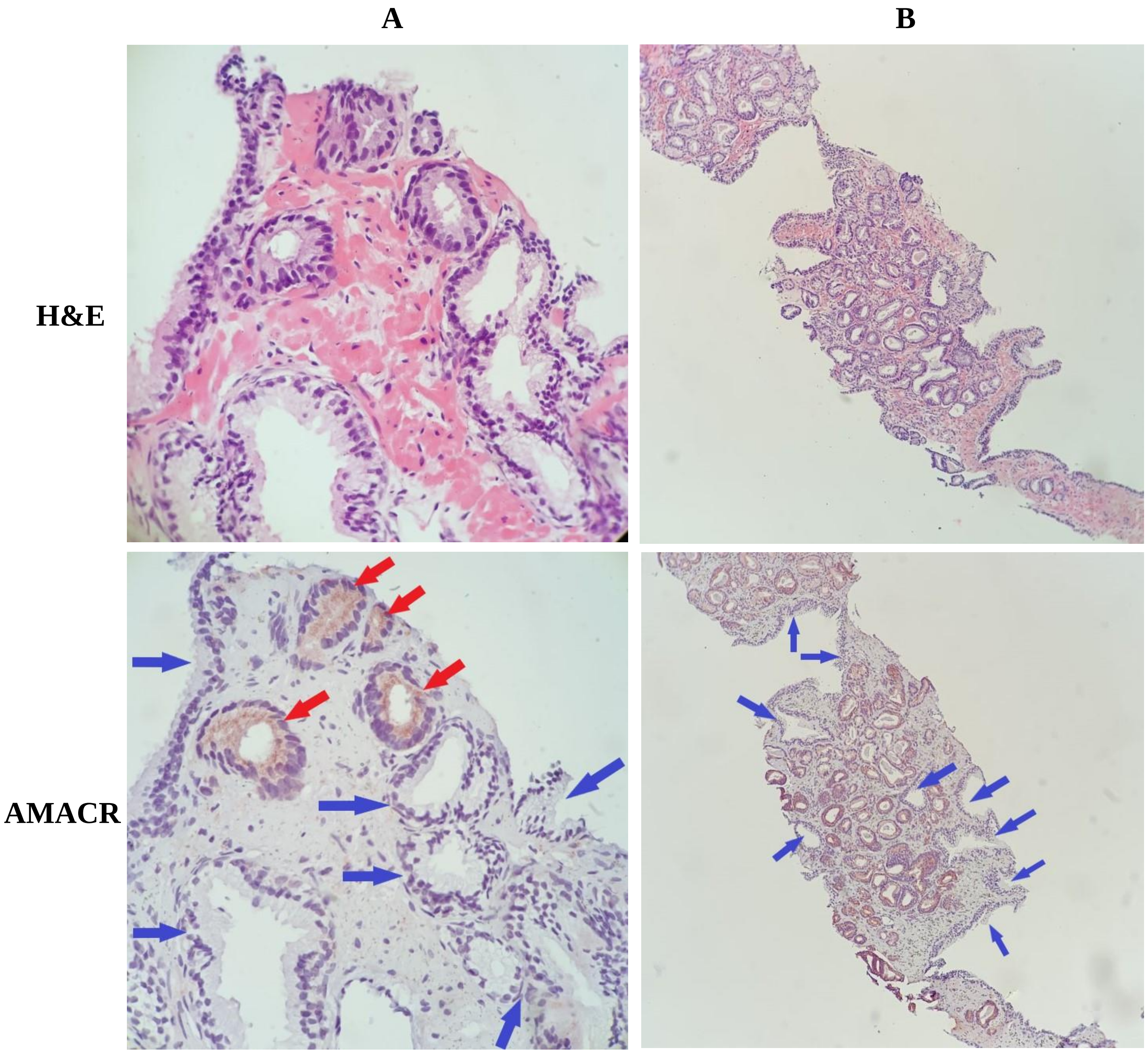

**Table 1s.** Correlation of **PSMA, GLUT1** and **ACLY** expression statuses in malignant epithelial cells with serum PSA level, tumor GS and time to progression.

| **PSMA** expression status in malignant component | | | |
|---|---|---|---|
| | Non overexpressing (28) | Overexpressing (6) | P-value |
| Mean serum PSA (at time of initial Dx) | 6.06 ± 0.44 | 8.16 ± 1.52 | 0.08 |
| Mean tumor GS | 6.29 ± 0.09 | 6.33 ± 0.21 | 0.82 |
| Mean time to progression (month) | 58.16 ± 8.90 | 21.65 ± 3.45 | 0.07 |
| **GLUT1** expression status in malignant component | | | |
| | Non overexpressing (21) | Overexpressing (13) | P-value |
| Mean serum PSA (at time of initial Dx) | 6.06 ± 0.64 | 7.03 ± 0.61 | 0.31 |
| Mean tumor GS | 6.19 ± 0.09 | 6.46 ± 0.14 | 0.0973 |
| Mean time to progression (month) | 63 ± 11.65 | 33.49 ± 4.32 | 0.06 |
| **ACLY** expression status in malignant component | | | |
| | Non overexpressing (19) | Overexpressing (15) | P-value |
| Mean serum PSA (at time of initial Dx) | 5.57 ± 0.59 | 7.64 ± 0.65 | 0.02 |
| Mean tumor GS | 6.22 ± 0.10 | 6.4 ± 0.13 | 0.28 |
| Mean time to progression (month) | 52.35 ± 9.41 | 47.88 ± 13.34 | 0.78 |
